# Evaluation of the taxonomic accuracy and pathogenicity prediction power of 16 primer sets amplifying single copy marker genes in the *Pseudomonas syringae* species complex

**DOI:** 10.1111/mpp.13337

**Published:** 2023-05-03

**Authors:** Chad Fautt, Kevin L. Hockett, Estelle Couradeau

**Affiliations:** ^1^ Department of Plant Pathology and Environmental Microbiology Pennsylvania State University University Park Pennsylvania USA; ^2^ Department of Ecosystem Science and Management Pennsylvania State University University Park Pennsylvania USA; ^3^ Intercollege Graduate Degree Program in Ecology Pennsylvania State University University Park Pennsylvania USA

**Keywords:** classification, effector proteins, PCR, *Pseudomonas syringae*, type III secretion system, virulence factors

## Abstract

The *Pseudomonas syringae* species complex is composed of several closely related species of bacterial plant pathogens. Here, we used in silico methods to assess 16 PCR primer sets designed for broad identification of isolates throughout the species complex. We evaluated their in silico amplification rate in 2161 publicly available genomes, the correlation between pairwise amplicon sequence distance and whole genome average nucleotide identity, and trained naive Bayes classification models to quantify classification resolution. Furthermore, we show the potential for using single amplicon sequence data to predict type III effector protein repertoires, which are important determinants of host specificity and range.

## Graphical abstract

In silico performance of popular *Pseudomonas syringae* species complex primer sets varies, but the best‐performing primers can provide high‐resolution classification and accurate virulence gene prediction.

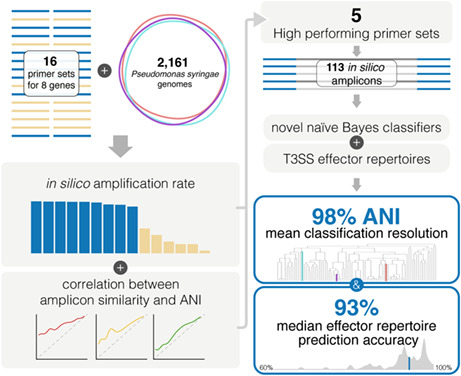

The *Pseudomonas syringae* species complex (PSSC) consists of many closely related plant pathogens (Sarkar & Guttman, 2004). With host ranges and symptomology that can overlap, accurate identification of isolates can be difficult (Morris et al., 2019). Aside from whole‐genome sequencing, which is costly and often impractical for routine identification, marker gene sequencing is the most effective method for specific and subspecific classification of unknown PSSC isolates (Berge et al., 2014; Borschinger et al., 2016; Guilbaud et al., 2016). This method has been used to aid in the identification of new pathogenic strains and species within the species complex, and to detect known pathogens infecting novel hosts (Dutta et al., 2018; Keshtkar et al., 2016; Moretti et al., 2012), highlighting the importance of amplicon sequencing for broadening our understanding of the species complex. However, although there have been many proposed PCR primer sets designed to amplify broadly within the species complex (Hwang et al., 2005; Sarkar & Guttman, 2004; Yan et al., 2008), there are open questions about the relative performance of each. Specifically, it is not clear if all primers allow for reliable amplification for all phylogroups in the species complex, or which primer sets allow for the greatest classification resolution. Furthermore, while a primary goal of pathogen identification is often to predict the pathogenic potential of the unknown isolate, it is not known if the classification resolution obtained by any of the currently published primers is sufficient to predict the genomic features associated with host range and virulence.

While most of the primer sets evaluated in this study were originally designed for use with multilocus sequence typing (Sarkar & Guttman, 2004; Yan et al., 2008), there has been continuous interest in classifying isolates with a single marker gene. In this regard, recombination rates and phylogenetic congruence have been used as metrics to suggest that genes such as citrate synthase (*CTS*) (Berge et al., 2014) and RNA polymerase σ factor (*rpoD*) (Parkinson et al., 2011) can be used by themselves to accurately place unknown PSSC isolates into phylogroups. An in‐depth comparison of these primer sets as tools for classification has not been conducted, however, leaving it an open question as to which performs best.

Often, an implicit goal of bacterial isolate identification is to gain some insight into its functionality or ecological significance for the environment it was isolated from. In this vein, assuming a phylogenetic placement with sufficient resolution, prediction of an isolate's gene content can be made, providing insight into functional capacity. This concept has been demonstrated with PICRUSt2, in which improved functional predictions were achieved over PICRUSt1 solely from increased resolution in genome prediction (Douglas et al., 2020). As PCR primer sets designed specifically for PSSC are used because they offer greater phylogenetic resolution over those targeting 16S rRNA genes (the marker gene used by PICRUSt2), we hypothesized that specific genes known to affect host range and virulence could be predicted in genomes based solely on amplicon sequences derived from commonly used PCR primer sets.

In PSSC, pathogenicity is determined in large part by the type III effector proteins (T3Es) carried by the pathogen (Hulin et al., 2018) and therefore predicting T3E repertoires could provide valuable information about the potential host range and specialization of an unknown isolate. While many pathogens in the species complex carry 30–40 T3Es, only effectors *avrE*, *hopM*, and *hopAA* are considered part of the core PSSC genome of PSSC and are thought to confer general virulence to plants (Dillon et al., 2019). The other T3Es play a role in host adaptation and are more variable in the species complex, suggesting that if there is a taxonomic signature associated with their presence, an infraspecific classification is needed to accurately predict it. It is currently not known what phylogenetic resolution is needed to accurately predict T3E repertoires or whether any published primer sets might allow high enough resolution to meet this threshold.

In the present study, we performed in silico tests to assess the performance of 16 previously published PCR primer sets, targeting eight marker genes, against 2161 PSSC genomes. The metrics used for assessment of phylogenetic classification were amplification rate, congruence of pairwise amplicon distance with average nucleotide identity (ANI), and performance of naive Bayes classifiers trained on in silico amplicon data. We also investigated the potential for functional prediction from amplicon data by analysing the Jaccard similarity of T3E repertoires at the level of phylogenetic resolution achieved by each classifier and show that isolates not included in the training dataset can be accurately placed above phylogroup level and prediction of both T3E repertoire size and content is often possible, with presence/absence of 77 T3E subfamilies being correctly predicted with a median accuracy of 93% among 113 genomes in a test dataset consisting of recently sequenced PSSC genomes.

Overall, we found that some published primer sets may have substantial blind spots in the lineages they can amplify. However, many primers tested could both amplify broadly throughout the species complex and be used to classify isolates beyond the phylogroup level, allowing accurate prediction of the T3Es carried by unknown PSSC isolates. Our results suggest that for the highest classification resolution throughout the species complex, resulting in the most consistent T3E repertoire prediction accuracy, primer sets targeting the genes *gapA*, *gyrB* (Hwang et al., 2005), and *PGI* (Yan et al., 2008) should be considered as the optimal primer sets.

A total of 2467 genomes labelled as belonging to the *Pseudomonas syringae* group were obtained from the RefSeq database from the National Center for Biotechnology Information in November 2021. Genomes were checked for completeness and assembly quality with BUSCO v. 5.3.2 using default settings and the pseudomonadales_odb10 lineage. Genomes scoring >99 made up the final dataset used for assessing primers. As the majority of genomes used were not assigned to a phylogroup, phylogroups were assigned based on ANI with phylogroup reference genomes produced by Berge et al. (2014). While Berge et al. suggest taking a simple nearest‐neighbour approach to assigning the phylogroup, 173 genomes within our dataset shared <95% ANI with any phylogroup reference genome, indicating that they were either misclassified at the time of depositing into GenBank as belonging to PSSC, or that they might represent new phylogroups. As a result, these genomes were left unassigned to a phylogroup. Eventually a curated set of 2161 genomes was used, with 1988 assigned to a phylogroup (Table S1 contains the accession numbers of these genomes and assigned phylogroups, along with ANI clusters and the T3E gene content described below).

In silico PCR was performed with ‘in_silico_PCR’ (Ozer, 2022) allowing for one mismatch per primer. The identity of amplicons was confirmed by visually inspecting multiple sequence alignments performed with MAFFT, using Geneious v. 2019.1.3 (https://www.geneious.com). The amplification rate reported is the percentage of the 2161 genomes that resulted in successful amplification of the target gene fragment. The primers included in this study are given in Table 1.

**TABLE 1:** Primer sets used in this study.

Primer set abbreviation (this paper)	Forward sequence (5′–3′)	Reverse sequence (5′–3′)	Original primer names	Source
gapA‐H	TCGARTGCACSGGBCTSTTCACC	GTGTGRTTGGCRTCGAARATCGA	gapA+312s/gapA−874ps	Hwang et al. (2005)
gyrB‐H	TCBGCRGCVGARGTSATCATGAC	TTGTCYTTGGTCTGSGAGCTGAA	gyrB+271ps/gyrB−1022ps	Hwang et al. (2005)
CTS‐H	CCTGRTCGCCAAGATGCCGAC	CGAAGATCACGGTGAACATGCTGG	gltA+513s/gltA−1130s	Hwang et al. (2005)
rpoD‐H	GYGAAGGCGARATYGRAATCG	CCGATGTTGCCTTCCTGGATCAG	rpoD+364s/rpoD−1222ps	Hwang et al. (2005)
CTS‐SG	CCCGTCGAGCTGCCAATWCTGA	ATCTCGCACGGSGTRTTGAACATC	cts‐Fs/cts‐Rs	Sarkar and Guttman (2004)
gapA‐SG	CGCCATYCGCAACCCG	CCCAYTCGTTGTCGTACCA	gapA‐Fps/gapA‐Rps	Sarkar and Guttman (2004)
gyrB‐S	MGGCGGYAAGTTCGATGACAAYTC	TRATBKCAGTCARACCTTCRCGSGC	gyr‐F/gyr‐R	Sawada et al. (1999)
rpoD‐S	AAGCGTATCGAAGAAGGCATYCGTG	GGAACWKGCGCAGGAAGTCGGCACG	rpo‐F/rpo‐R	Sawada et al. (1999)
acnB‐Y	TGATGTTTGATGCCTTCCAC	TAAAACCCTTGGTGCTTTCG	acnB	Yan et al. (2008)
Gap1‐Y	CGTATCGCAATCAACGGTTT	GACTCTCCGTATCGCAATCA	gap‐1	Yan et al. (2008)
CTS‐Y	WYTRACCGGYACMGTBGGY	TGGGCTGATSGGYTTRATYT	gltA	Yan et al. (2008)
gyrB‐Y	TGCVTTCGTTGARTACCTGA	ACGGAAGAAGAAGGTSAGCA	gyrB	Yan et al. (2008)
PGI‐Y	GCGTACTACCGYAMYCCBTC	CCACATMGGRAARATRTTYT	pgi	Yan et al. (2008)
rpoD‐Y	GAAGGCATCCGTGAAGTGAT	GCCACGGTTGGTGTACTTCT	rpoD	Yan et al. (2008)
rpoB‐T	TGGCCGAGAACCAGTTCCGCGT	CGGCTTCGTCCAGCTTGTTCAG	LAPS/LAPS27	Tayeb et al. (2005)
rpoD‐P	TGAAGGCGARATCGAAATCGCCAA	YGCMGWCAGCTTYTGCTGGCA	PsrpoDFNP1/PsrpoDnprpcr1	Parkinson et al. (2011)

Pairwise ANI values for all genomes were computed with fastANI v. 1.33 (Jain et al., 2018). For each primer set with amplification rate >50%, amplicon sequences were aligned using MAFFT v. 7 (Katoh & Standley, 2013) with the options ‘globalpair’ and a ‘maxiterate’ of 1000. For amplicon sequence similarity, pairwise hamming distances for the aligned sequences were then computed with the ‘DistanceMatrix’ function in the R package DECIPHER (Wright, 2015). To quantify the correlation between amplicon sequence distances and ANI of the source genomes, the mean squared deviation of amplicon sequence similarity from ANI was computed as the sum of squared distances between the two values for each genome pair. As ‘DistanceMatrix’ reports distance in the range of 0–1, ANI values were normalized to the same range by dividing by 100.

Using QIIME2's v. 2022.2 feature‐classifier (Bolyen et al., 2019), naive Bayes classifiers were trained on the unaligned in silico amplicon sequences generated above. Primer sequences were left untrimmed from amplicons. Classification models require taxonomic descriptions of known genomes for training, but nomenclature in PSSC is largely inconsistent (Gomila et al., 2017), which can significantly reduce the predictive power of classification models. We therefore implemented instead a strict hierarchical taxonomy based on ANI, generated by the clustering algorithm used by LINbase (Tian et al., 2020). Briefly, a randomly selected genome in the set is assigned to clusters representing 20 ANI values from 80% to 99%, each given a numeric signature of ‘0’. All other genomes are iteratively assigned to clusters based on the closest already‐assigned genome, and given the same numeric signature for all ANI values up to the point that the two genomes differ, wherein the numeric signature is iterated (e.g., if genome #2 shares 97.5% ANI with genome #1, genome #2 will be assigned to cluster ‘0’ along with genome #1 for all ANI values except 98%, where it will be assigned a numeric signature of ‘1’). The resulting taxonomy file consists of 20 taxonomic levels representing ANI values from 80% to 99% (Table S1).

Reference sequences for T3E subfamilies included in the *P. syringae* type III effector compendium (PsyTEC) (Laflamme et al., 2020) were aligned using MAFFT with default settings, and the alignments input into the HMMER v. 3.1b2 (hmmer.org) function HMMbuild to generate HMM profiles. Using HMMsearch, these 77 HMMs were run on the set of 2161 genomes and an e‐value of 10^−20^ was used as the threshold for considering a subfamily to be present in a genome.

As all primers used in this study were designed to work broadly on strains within the species complex, we first tested the amplification rate of each primer set. Surprisingly, when tested on a comprehensive set of genomes representing the full known diversity of PSSC, 7/16 primer sets tested had an amplification rate of <50% (Figure 1a). For the remaining nine primers, performance was substantially better, with amplification rates ranging from 91.37% (rpoD‐P) to 100% (rpoD‐H). These large differences in amplification rate are probably due to the significant degeneracy built into the best performing primer sets (Tables 1 and S2) and highlights the importance of considering the known diversity of PSSC when designing primers. It is worth noting, however, that the low in silico amplification rates seen here do not necessarily translate to low amplification rates under laboratory conditions, as only a single mismatch was allowed per primer in our tests. In practice, successful amplification with two or more mismatches is not unreasonable to expect. Nonetheless, as primer sets with fewer mismatches are generally preferred, and the majority of the primer sets exhibiting low amplification rates targeted genes already targeted by better performing primer sets, primer sets with amplification rates <50% were removed from any further tests.

**FIGURE 1:**
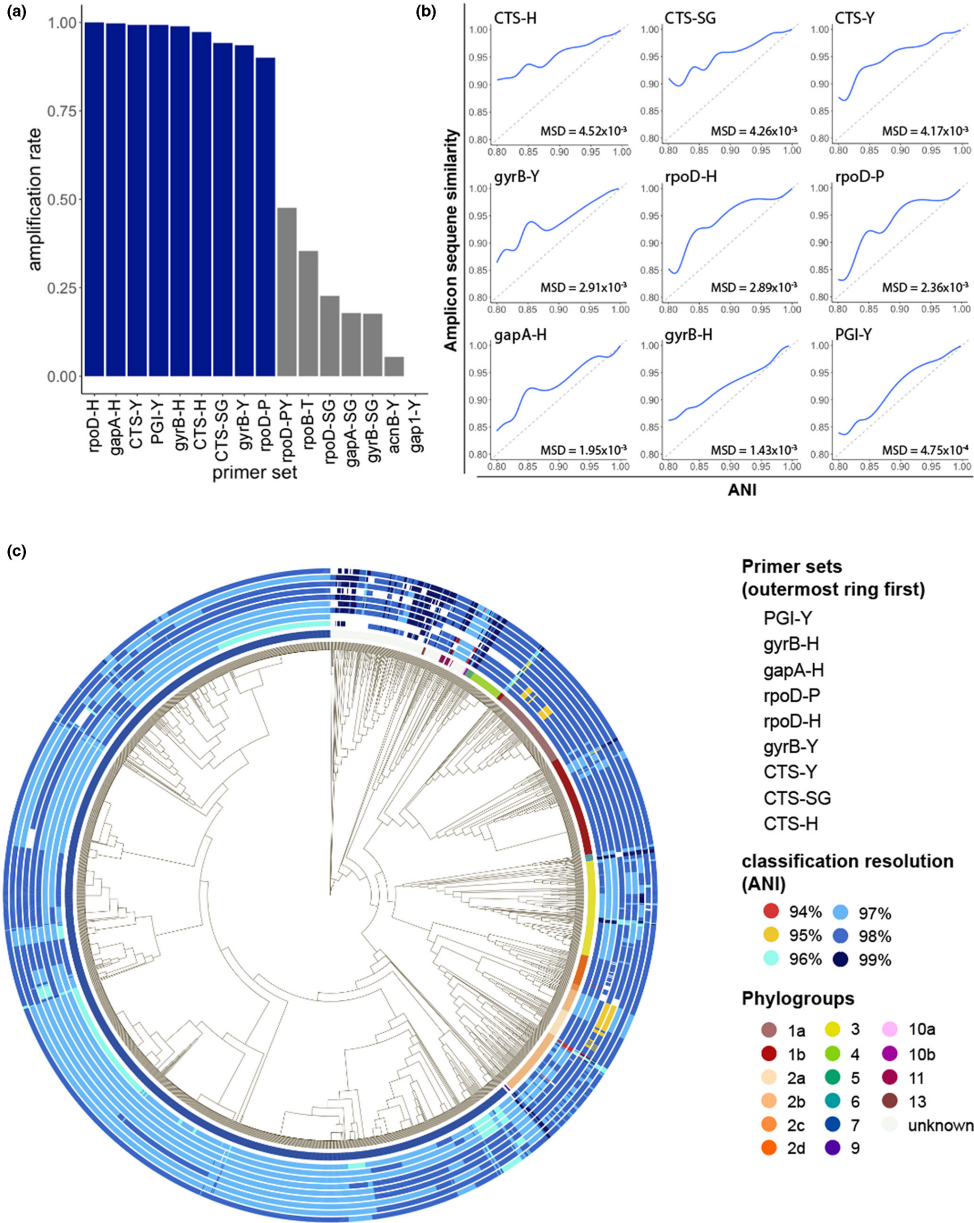
Results of amplicon‐based classification tests. (a) Proportion of genomes with successful amplification, allowing one mismatch per primer. Primer sets producing amplicons in more than 90% of genomes are highlighted in blue. Primer sets in grey were omitted from further analysis. (b) Generalized additive models summarizing the relationship between pairwise amplicon similarity and whole‐genome average nucleotide identity (ANI). Mean squared deviation (MSD) of amplicon similarity from ANI is shown in the lower left corner and the dashed grey line represents MSD = 0. (c) Core genome phylogeny for all genomes used in this study. Innermost ring annotates phylogroups, where each outer ring represents the classification resolution obtained when amplicon sequences from each genome were identified with a Bayes classifier. White rectangles represent genomes from which in silico amplification was unsuccessful.

When choosing a marker gene, or region of a marker gene, to use for identification purposes, an important consideration is the level of conservation found within the region, as regions that are too conserved result in reduced taxonomic resolution. To investigate the amount of conservation found within the amplicons generated by each primer set, we compared pairwise amplicon similarity (represented by their Jaccard index) with the ANI of the genomes from which the amplicons were derived (Figure 1b). Of the primers tested, amplicons from *CTS* showed the highest level of conservation, indicating they might not provide the best resolution when used for classification contrary to previous suggestions (Berge et al., 2014). On the other hand, amplicons generated by PGI‐Y, targeting phosphoglucose isomerase (*PGI*), exhibited a mean squared deviation from ANI almost 10 times lower than any primer targeting *CTS* (Figure 1b), indicating a very good congruence between the diversity at this locus and the one retrieved at the genome level.

To compare the performance of primers in classifying individual strains throughout PSSC, classification models were trained on amplicons generated by each primer set and then used to classify each strain in the training set. The relative performance of the classification models mirrored the amount of conservation observed above (Figure 1b), although the practical differences in classification resolution were minimal (Table 2). Remarkably, every primer set allowed for classification beyond phylogroup level, with the mean ANI of predicted clades ranging from 97.22% (CTS‐H, CTS‐Y, and CTS‐SG) to 97.93% (PGI‐Y). Surprisingly, while the *CTS* gene has been suggested to be a particularly informative marker gene for PSSC (Berge et al., 2014), the three primers targeting this gene performed slightly below the other primers tested. Although the mean performance of classification models suggests PGI‐Y as the best primer set, it does not consider biases in the representation of each phylogroup in our dataset, and so we sought to analyse any discrepancies in primer performance among phylogroups (Figure 1c).

**TABLE 2:** Summary of classification test results.

Primer set	Mean average nucleotide identity resolution	*SD*
gyrB‐H	97.5970987	0.5956094
CTS‐SG	97.2226044	0.5965339
CTS‐Y	97.2277597	0.6516167
gapA‐H	97.5547818	0.5947916
rpoD‐P	97.6722441	0.6654118
PGI‐Y	97.9319664	0.4392588
gyrB‐Y	97.5712166	0.5701288
rpoD‐H	97.6307265	0.6961651
CTS‐H	97.2234903	0.8056386

For strains belonging to phylogroup 1, all primers performed well, amplifying every strain tested and classifying most to 98% ANI (Figure 1c). An exception to the strong performance can be seen for two subclades which CTS‐Y and CTS‐SG were only able to classify at 95%. Overall, the best performing primers for phylogroup 1 were gapA‐H and gyrB‐H.

In phylogroup 2, performance was more variable. Both rpoD‐P and rpoD‐H were unable to classify strains in a well‐sampled clade of phylogroup 2b above 95% ANI, and gyrB‐Y was unable to amplify several strains within phylogroup 2d. As seen in phylogroup 1, the best performing primers for phylogroup 2 were gapA‐H and gyrB‐H.

Phylogroup 3 strains were successfully amplified by every primer, with the exception of four strains that gyrB‐H was unable to amplify from. Overall, PGI‐Y and gyrB‐Y were the best‐performing primers for strains in phylogroup 3.

All primers performed equally well for phylogroup 4 strains, classifying to 98% ANI. gyrB‐Y, however, failed to amplify from every strain in this phylogroup.

Our dataset contained only nine phylogroup 5 strains, but every primer set was able to amplify and classify each one to 98%–99% ANI.

As with phylogroup 4, gyrB‐Y was the only primer set unable to amplify and classify to 97%–99% every strain in phylogroup 6.

Within phylogroup 7, PGI‐Y performed the best, classifying 89% (1189/1331) of strains to 98% ANI, while all other primers generally classified strains in this phylogroup to 97%.

Phylogroups 9, 10, 11, and 13 were under‐represented in the dataset and so conclusions are difficult to draw about primer performance. Additionally, there were several strains not assigned to a phylogroup in our dataset. Among these strains, rpoD‐H and gapA‐H exhibited the highest amplification rates and classification resolution ranging from 97% to 99% ANI.

No single primer set universally outperformed the rest, and as such the suspected identity of an unknown isolate should be considered when choosing the appropriate set of primers to use for classification. However, PGI‐Y, gapA‐H, and gyrB‐H generally performed well throughout the species complex and should be considered the default choices for the highest classification resolution.

As classification based on single amplicon sequences resulted in fairly high genomic resolution (97%–98% ANI), we sought to explore the possibility of predicting the gene content of a target strain using the gene content of predicted relatives. As T3Es are important determinants of virulence in PSSC (Dillon et al., 2019; Lindeberg et al., 2009), we focused here on T3Es by first assessing the prevalence of each T3E subfamily within genomic clusters sharing at least 98% ANI (Figure 2). While there were several clusters that contained only a single genome (i.e., no genome in the dataset shared more than 98% ANI with them), even among better represented clusters, there was considerable similarity in T3E repertoires. This suggested that unknown isolates placed into these clusters should exhibit a predictable T3E repertoire. Perhaps not surprisingly, clusters representing phylogroups that contain most of the agricultural pathogens within PSSC exhibit many more T3Es as well as a greater diversity in repertoires between strains, indicated by the average Jaccard index within a given cluster (Figure 2). When the T3E repertoires of the 2161 genomes were compared against the consensus repertoires (defined by taking the most common state of each T3E subfamily, absent or present) of their 98% ANI clusters, 75.3%–100% of actual T3E states recapitulated the intracluster consensus (Figure 3). It should be noted that the T3E repertoires in our analysis were defined solely on the presence or absence of gene subfamilies, and that even single amino acid changes within effector protein sequences can alter host compatibility. Therefore, the T3E repertoire predictions presented here should be considered a useful starting point for generating hypotheses regarding the host range of unknown isolates, and not any prediction of host range in itself.

**FIGURE 2:**
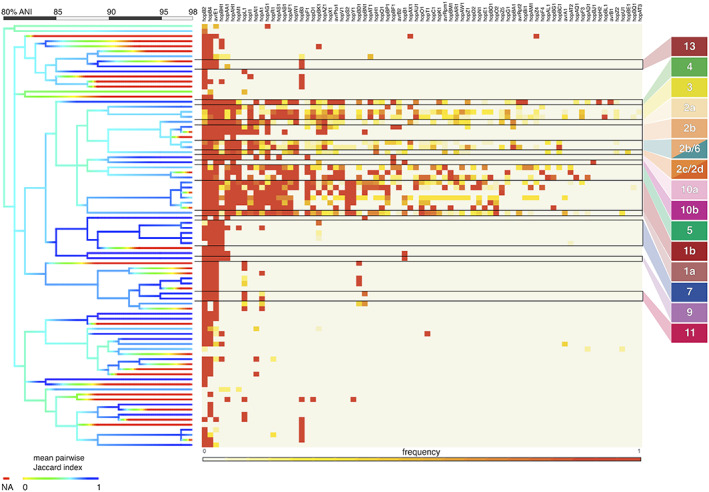
Distribution of type III effector proteins throughout the *Pseudomonas syringae* species complex. The heatmap shows the frequency of each type III effector subfamily, with each row as a cluster of genomes sharing 98% average nucleotide identity (ANI). Black outlines indicate the phylogroups found in each row. The cladogram on the left represents ANI‐based clusters of genomes from 80% to 98% ANI and is coloured by the mean Jaccard index of genome pairs found in each branch. Red branches indicate singletons for which the Jaccard index could not be calculated.

**FIGURE 3:**
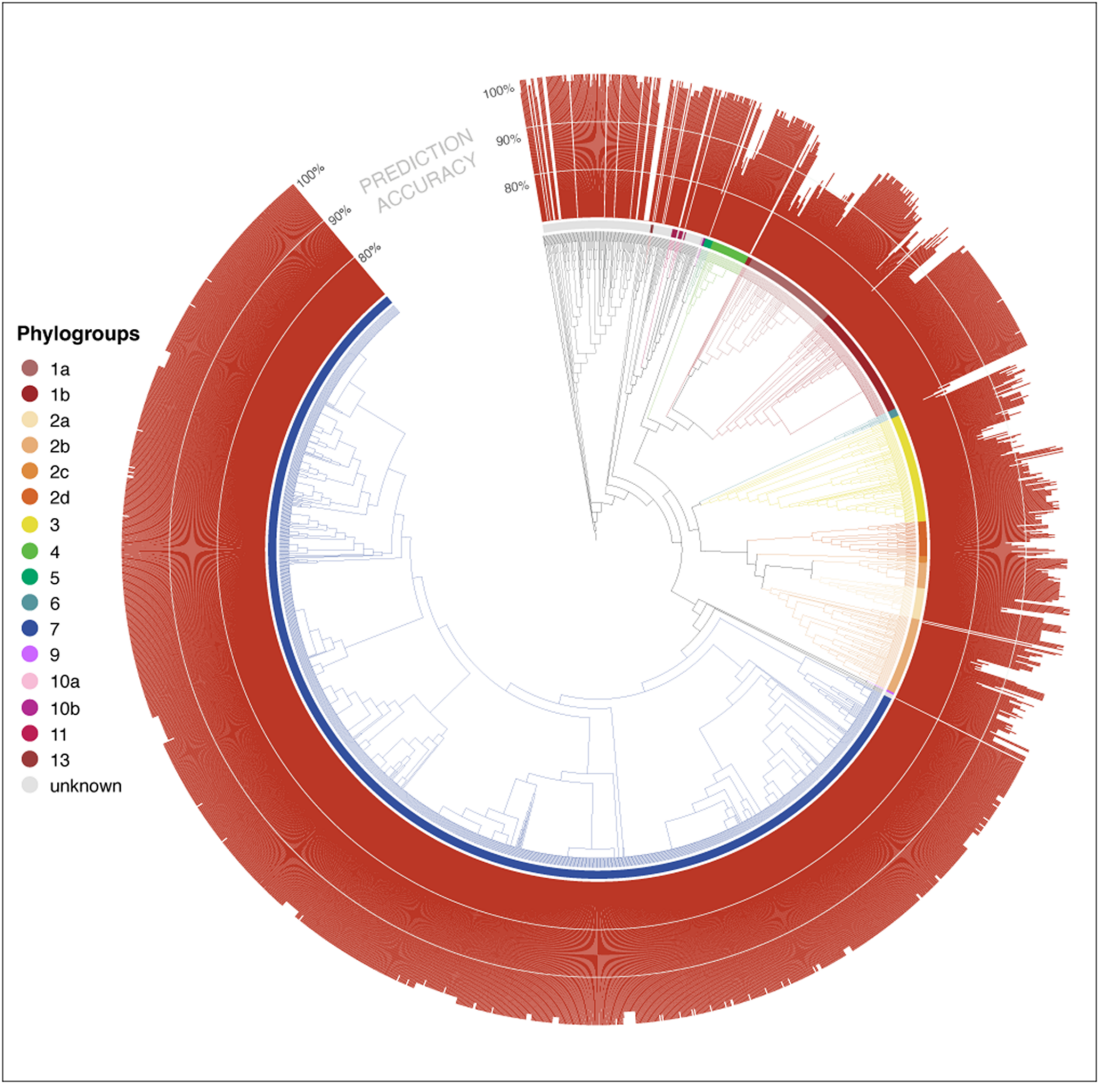
Similarity between individual type III effector repertoires and the consensus repertoire at the 98% average nucleotide identity level. The height of the bar represents percentage agreement, scaled from 70% to 100%. Genomes with no bar represent singletons at the 98% level, and thus no consensus repertoire could be calculated. Innermost ring colours designate phylogroups, as seen in Figure 1.

To further test the feasibility of T3E repertoire prediction, 113 genomes not included in our initial training set underwent in silico PCR for the nine primers tested above, classified using the trained naive Bayes classifiers and screened for T3Es. Actual T3E repertoires were then compared to the average repertoire within the predicted LIN group of the unknown strain and prediction accuracy for the repertoire was assessed. While not every primer set was able to amplify from every genome (Figure 4a), amplification rates were generally good at 92.9%–100%. Classification of genomes from amplicon sequences resulted in the placement of most strains to 98% ANI or greater (Figure 4a). The overall accuracies of T3E repertoire predictions for all primers were nearly identical; each primer set allowed for a median prediction accuracy of 93.51% (Figure 4b) but with individual prediction accuracies ranging widely from 64.9% to 100%. As some phylogroups are known to contain a greater number and diversity of effector proteins, we asked whether prediction accuracy varied significantly by phylogroup and found a strong correlation between phylogroup and prediction accuracy (Figure 4c). We also found that some T3Es were particularly difficult to predict (Figure 4d) using our method. For example, for *hopA1*, a predicted absence of the subfamily was a false negative more often than not. Likewise, *avrE1* was predicted to be present 100% of the time in our test genomes, resulting in false positives in 27% of genomes. These findings strongly suggest that, at a minimum, any implementation of gene content prediction based on classification from marker gene sequences needs to consider the appropriate testing errors to be of practical use.

**FIGURE 4:**
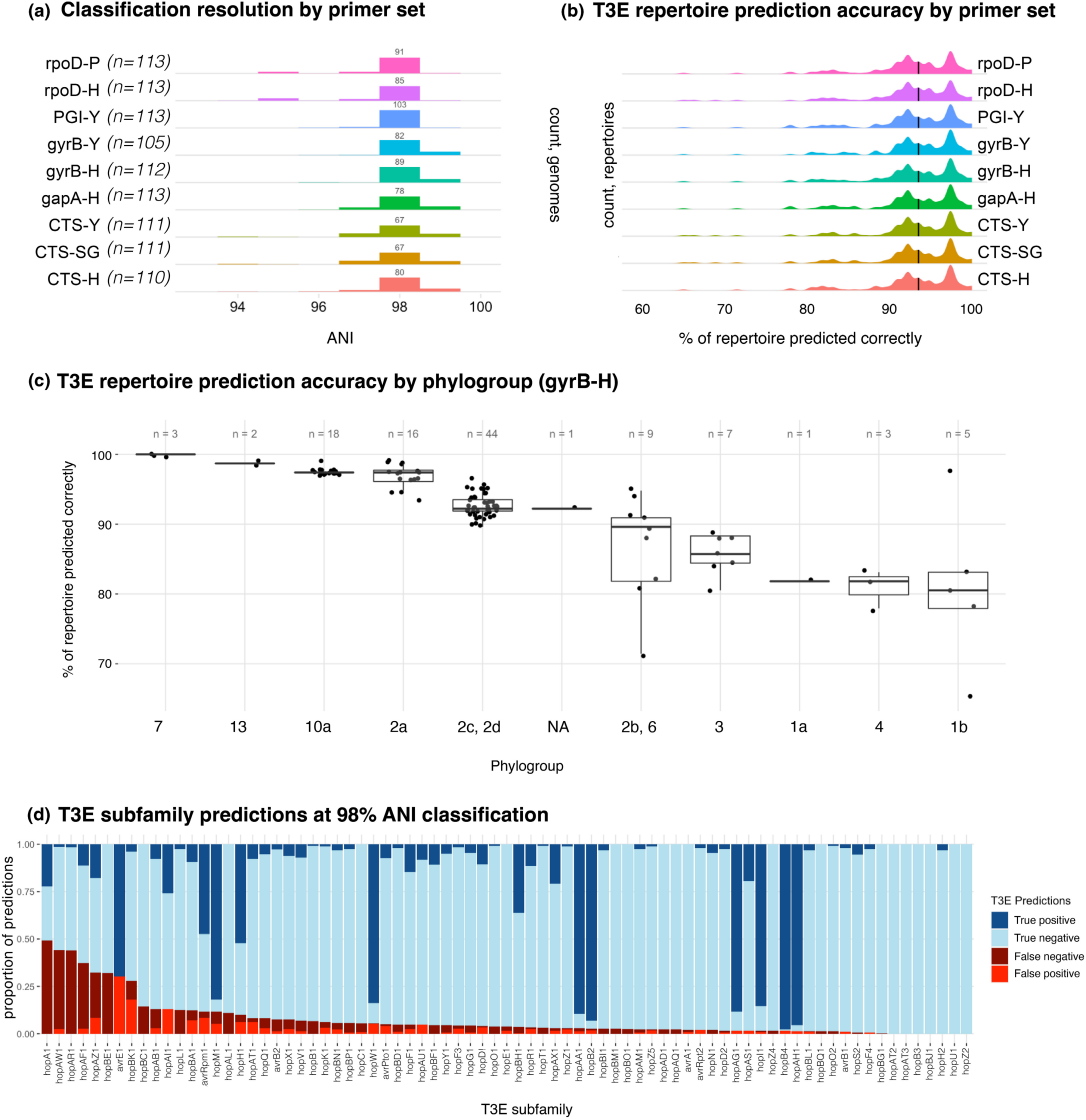
Classification based on single marker genes allows for accurate prediction of type III effector (T3E) repertoires. (a) Density plots for classification resolution for each primer set. The number of successful in silico amplifications from each primer set, out of 113, is indicated in parentheses. (b) Density plots for the T3E repertoire prediction accuracy for each primer set. Prediction accuracy is defined as the percentage of 77 effector protein subfamilies whose absence or presence in a genome was predicted correctly. (c) Boxplots for T3E repertoire prediction accuracy by phylogroup of the classified genome. Each dot is a single genome whose T3E repertoire was predicted based on classification with primer set gyrB‐H. (d) Summary of T3E subfamily predictions in test genomes classified to at least 98% average nucleotide identity, sorted from least to most correct predictions.

As more PSSC genomes are sequenced and deposited in public repositories, it is likely that our ability to predict T3E repertoires, as well as the presence of other important virulence factors, from amplicon sequencing will improve. This, coupled with research that indicates that host range can in part be inferred from virulence factors such as T3Es (Baltrus et al., 2012; Ferrante et al., 2009; Hulin et al., 2018) suggests that amplicon sequencing remains a powerful method for studying disease dynamics, predicting pathogen spread, and rapidly detecting problematic PSSC strains.

In this study we set out to compare PCR primer sets designed to amplify broadly within the PSSC. We found that there were significant differences in amplification rates that raise questions about the utility of some commonly used primers. However, we also found that classification resolution was relatively consistent between the primers tested, allowing placement of unknown genomes into clusters at the 98% ANI level.

The high resolution obtained from our classification models led us to investigate the potential of single amplicon sequences for the prediction of T3E protein subfamilies. We showed that with median accuracy of 93%, we were able to correctly predict the effector repertoires of 113 recently sequenced PSSC strains, although the accuracy was dependent on phylogroup. These results highlight the importance of continued isolation and sequencing of plant pathogens as a source of data to be leveraged in the future for more efficient and informative screening assays. Based on our findings here, we currently recommend the primer sets gapA‐H, gyrB‐H, and PGI‐Y for single amplicon sequence typing of isolated PSSC strains.

## CONFLICT OF INTEREST STATEMENT

The authors have no conflicts of interest to declare.

## Supporting information


**Table S1.** Data associated with genomes used in this study. Includes assigned phylogroups, ANI‐based cluster assignments, and accession numbers and metadata extracted from GenBank.

**Table S2.** Amplification rate for each primer set tested, by phylogroup.

## Data Availability

T3E sequences detected by HMMER and used to assess repertoire content are available on Zenodo at 10.5281/zenodo.7249289. In silico PCR results for primer sets exhibiting >50% amplification rate are available on Zenodo at 10.5281/zenodo.7249269. All other data are available on request from the corresponding author.
